# Uncertainty management strategies in clinical reasoning: perceptions of nurses in post-anesthesia care units

**DOI:** 10.1186/s12912-025-03193-8

**Published:** 2025-05-28

**Authors:** Lara Daniela Matos Cunha, Paul K. J. Han, Filipa Ventura, Márcia Pestana-Santos, Lurdes Lomba, Margarida Reis Santos

**Affiliations:** 1 Health Sciences Research Unit: Nursing (UICISA: E), Coimbra Nursing School, Coimbra, Portugal; 2 School of Medicine and Biomedical Sciences (ICBAS) - University of Porto (UP), Porto, Portugal; 3 Foundation for Science and Technology (FCT), Lisboa, Portugal; 4 Division of Cancer Control and Population Sciences, National Cancer Institute, Maine, USA; 5 Coimbra Nursing School (ESEnfC), Coimbra, Portugal; 6 Nursing School of Porto (ESEP), Porto, Portugal; 7 Center for Health Technology and Services Research (CINTESIS@RISE), Porto, Portugal; 8 Health Sciences Research Unit: Nursing (UICISA: E), Coimbra Nursing School (ESEnfC) - Pólo C, Avenida Bissaya Barreto, Coimbra, 3046-851 Portugal

**Keywords:** Clinical reasoning, Nursing realities, Patient safety, Post-anesthesia nursing, Uncertainty

## Abstract

**Background:**

While it is acknowledged that effectively managing uncertainty in nursing clinical practice is imperative for ensuring safe healthcare delivery, there remains a scarcity of guidance on enhancing uncertainty management within the clinical reasoning of nurses.

**Aims:**

To describe strategies that nurses in post-anesthesia care units use to manage uncertainty in clinical reasoning; and to map these strategies within the Integrative Framework of Uncertainty Management, and the clinical realities of Wiedenbach’s Prescriptive Theory.

**Material & methods:**

A descriptive qualitative study. Fourteen nurses from a post-anesthesia care unit of a third-level hospital at the center region of Portugal were recruited using a convenience sampling method. Data were collected through semi-structured interviews and analyzed using thematic analysis using MAXQDA software. Consolidated Criteria for Reporting Qualitative Research (COREQ) checklist was used for reporting. Ethical approval was obtained from the relevant Ethics Committee, with informed consent, confidentiality, and data protection measures in place to ensure participant anonymity and compliance with the Declaration of Helsinki.

**Results:**

The major theme ‘uncertainty management strategies in clinical reasoning of nurses in Post-anesthesia Care Units’ included four themes: ignorance-focused strategies, uncertainty-focused strategies, response-focused strategies, and person-focused strategies. These themes were mapped within key domains of nursing care: recipient, skills, policies, techniques, time sequence, agent, happenings, and supportive relationships. The domains encompassed various approaches such as person-centered care, evidence-based practice, postgraduate training, conflict management, critical thinking, continuous improvement projects, in-service team training, contingency planning, case discussions, simulation, self-reflectiveness, emotion self-regulation, optimizing working conditions, positive cognitive reframing, seeking support, fostering team reflection, and collaborative communication.

**Conclusion:**

Nurses in the Post-anesthesia Care Unit employed a range of uncertainty management strategies, which were effectively categorized within the Integrative Framework of Uncertainty Management (IFUM). Emphasizing nursing as a process, as described in Wiedenbach’s Prescriptive Theory, these strategies were aligned with addressing the clinical realities tailored to each strategy. More research is needed to understand how the use of different strategies can promote nurses’ wellbeing and enhance the safety of nursing care.

**Clinical trial registration:**

Not applicable.

**Supplementary Information:**

The online version contains supplementary material available at 10.1186/s12912-025-03193-8.

## Introduction

Clinical reasoning is a fundamental process in nursing practice, wherein nurses utilize their knowledge, skills, and experience to assess patient conditions and make informed decisions regarding care [1]. It involves a dynamic, iterative process of collecting data, analyzing information, and making judgments, with the ultimate goal of ensuring safe and effective patient outcomes [2]. While clinical judgment and clinical reasoning are closely related, they serve distinct roles. Clinical judgment refers to the ability to make decisions in uncertain or ambiguous situations, whereas clinical reasoning encompasses the cognitive processes that guide decision-making in patient care [3]. However, clinical reasoning becomes even more complex in situations of uncertainty, where nurses must navigate incomplete information, unpredictable patient responses, and rapidly changing clinical conditions [4]. As a core competency in nursing [2], clinical reasoning is both dynamic and multifaceted, requiring nurses to continuously adapt to the dynamic and unpredictable nature of real-world clinical scenarios [5].

The complexities of the perioperative system, including workflow variability, diverse equipment handling, and ongoing uncertainty related to dynamic responses, contribute to the challenges faced in managing patient care [6]. Despite significant advancements in surgical safety, adverse event rates remain between 18% and 22%, with patient complications posing a major global public health challenge [7]. In resource-limited Post-Anesthesia Care Units (PACU), the incidence of complications can rise to 54.8% [8]. These challenges also resonate with complex decision-making in post-operative care [9], and uncertainty may present a significant barrier to effective clinical reasoning among nurses in PACU settings. The intricacies of uncertainty related with healthcare require a level of adaptability that traditional clinical reasoning frameworks may not always provide [10]. Therefore, describing the strategies that nurses in PACU use to manage uncertainty in clinical reasoning, and aligning them with specific frameworks and context-specific nursing clinical realities, can assist other nurses in better coping with the intrinsic uncertainties in post-anesthesia care practice [11].

Uncertainty is the metacognitive and conscious awareness of ignorance [12]. It emerges from a combination of external elements and circumstances in nursing care, which can shape the course of clinical events. Therefore, it is crucial to consider Wiedenbach’s Prescriptive Theory (PT) [13] that hinges on three pivotal components: foremost, the practitioner’s recognition of the central purpose deemed essential in the realm of nursing; secondly, the prescription detailing how to fulfill this central purpose; and notably, the immediate situational realities within the nursing context that exert a profound influence on achieving the central purpose. The realities of nursing practice encompass physical, physiological, psychological, emotional, and spiritual factors that need to be embedded in the nursing action [13]. These factors can enhance the ability of nurses to achieve the desired results, and patients’ ability to benefit from nursing care. However, this dynamic is unpredictable and disruptive, which nurses need to acknowledge and to respect, accept, and cope with consciously and responsibly. The ability to manage uncertainty is essential to accomplishing all of these tasks [13].

A study [14] offered practical tips for managing uncertainty, focused on oneself, students and trainees, and patients in health systems. Another study [15] investigated the strategies adopted by emergency nurses to address uncertainty in the event of emerging infectious diseases. Identified strategies included completion of a comprehensive assessment, continuing education, incorporation of guideline updates and mentoring into new roles and competences. The researchers also emphasized the need for education and training schemes that allow nurses to acquire and develop the necessary decision-making and problem-solving skills. This aligns with research [16], which found that sharing essential information for reviewing and developing clinical practice guidelines serves as a foundation for nursing care.

Uncertainty management entails surveying different approaches, as well as fostering uncertainty tolerance, which ultimately requires a comprehensive understanding of uncertainty. A conceptual framework of uncertainty management strategies developed by Han [17], hereafter referred to as the Integrative Framework of Uncertainty Management (IFUM), identifies interrelated tasks organized into four main categories according to their goals and focus, i.e. ignorance-focused, uncertainty-focused, response-focused, and person-focused. Ignorance-focused strategies aim to reduce uncertainty in clinical reasoning by decreasing the ignorance that constitutes its root cause. Uncertainty-focused strategies target not the ignorance that represents the object of one’s uncertainty, but the awareness of ignorance, which is necessary for uncertainty to exist as a metacognitive state. Response-focused strategies address people’s psychological responses to uncertainty. These strategies differ from the ones that focus on ignorance or uncertainty in that they aim to palliated—i.e., soften or mitigate—its aversive psychological effects, rather than to reduce or ‘’cure’’ uncertainty. Person-focused strategies are anchored on personal relationships, which may be both interpersonal and interprofessional [17].

The strategies employed to regulate uncertainty are intended to meet at least one of two main objectives: cure or palliation. Cure is the goal when clinical uncertainty is reducible, and palliation when uncertainty is irreducible. However, the reducibility and irreducibility of uncertainty can vary, according to the individual and situation [17]. Uncertainty management entails surveying different approaches, as well as fostering uncertainty tolerance, which ultimately requires a comprehensive understanding of uncertainty.

Strikingly, the empirical evidence on uncertainty management strategies used by nurses is limited, especially concerning information-seeking ones [18]. Identifying and systematically classifying uncertainty management strategies is a crucial first step toward developing comprehensive and accessible approaches to enhancing nurses’ ability to manage uncertainty. By addressing this gap, this study seeks to offer practical insights that can directly inform clinical practice, improve nursing competency, and ultimately lead to better patient outcomes in these critical care settings. The aim is two-fold: (1) to describe strategies that nurses in post-anesthesia care units use to manage uncertainty in clinical reasoning (such as ignorance-focused, uncertainty-focused, response-focused, and person-focused strategies); and (2) to map these strategies within the Integrative Framework of Uncertainty Management and the clinical realities described by Wiedenbach’s Prescriptive Theory.

## Materials and methods

### Research design

A qualitative descriptive study [19] was performed to capture PACU nurses’ perceptions of uncertainty management strategies in clinical reasoning.

### Setting and participants

The first author requested the nurse manager’s assistance in disseminating the research opportunity to the 20 eligible PACU nurses through an institutional email announcement. Nurses expressing interest subsequently reached out to the primary author, who conducted further screening to confirm their eligibility. Convenience sampling [20] was employed to select nurses providing postoperative care in Phase I [21] in the adult PACU. The Phase I PACU cares for patients directly after surgery, providing continuous nursing monitoring and addressing essential life-sustaining needs during the immediate postoperative recovery period [22]. The recruitment focused on a single PACU, managing an average of 30 to 35 surgeries daily and approximately 8.478 admissions annually. Post-anesthesia care was extended to various surgical specialties (colon and rectal, general, gynecology and obstetrics, gynecologic oncology, neurological and neurosurgery, ophthalmic, oral and maxillofacial, orthopedic, otorhinolaryngology, plastic and maxillofacial, urology, urogynecology and reconstructive pelvic surgery, vascular medicine) and nonsurgical specialties (dermatology, neuroradiology, psychiatry), along with special programs (kidney and liver transplantation, brain aneurysms) and a multidisciplinary approach to pain management. Nurses who were in training were excluded (less than six months of PACU professional practice and still in mentoring). Fourteen nurses who expressed interest in participating in the study were included and none dropped out.

### Data collection

Individual semi-structured interviews [23] were used to collect data on the studied phenomenon from the participants. After recruiting nurses, individual face-to-face semi-structured interviews were conducted in a private room at the anesthesiology service, following a moderator guide developed by the research team (Supplementary File 1). The guide covered topics including participants’ perspectives on problem-solving, solution identification, exploration of alternative strategies, evaluation of nursing intervention outcomes, and the resources used in managing uncertainty within the clinical reasoning of PACU nurses. We followed a structured approach [24] comprising three key steps: utilizing open-ended prompts to identify core events or experiences that illustrate the phenomenon of interest, structuring the questions to facilitate a natural and conversational flow - including an introductory rapport-building phase, a central exploratory section, and a final reflection to encourage additional insights - and refining the schedule through iterative review and piloting. A pilot interview [23] with one participant was conducted to assess the appropriateness of the questions, and no revisions were deemed necessary, making the pilot interview part of the final sample. Data collection took place from April to May 2022, involving 14 interviews lasting between 30 and 64 min, facilitated by the first study author (LC), a female medical-surgical nurse specialist and doctoral student working in the PACU. As insider researcher, LC was familiar with the local reality, maintained a reflexive approach during and after interviews, making annotations for clarification in subsequent sessions. All interviews were audio recorded and transcribed verbatim through manual transcription. Participant demographic information, including gender, age, qualifications, academic degree, and years of professional experience as a nurse (both globally and in the PACU), was collected. The data collection concluded when thematic saturation was achieved, and no new data emerged [25].

### Data analysis

In this study, the researcher LC served as both the interviewer and the transcriber, which helped minimize potential biases and ensured the quality of the transcript. The naturalized transcripts [26] were provided to each participant for validation [27], and all participants returned the interviews to the researcher without making any modifications. Data were analyzed hybrid inductive-deductively [28] following the IFUM and PT theoretical framework [13, 17] using Braun & Clark thematic analysis [29]. In Phase 1, researchers LC and MPS immersed themselves in the data to grasp its depth and breadth, identifying preliminary meanings and patterns. Phase 2 involved coding, wherein codes were generated based on the components of the theoretical framework, and data was organized accordingly. In Phase 3, data sets were coded and organized into themes using the coding guide derived from the theoretical frameworks. In Phase 4, a hybrid inductive-deductive analysis approach was used to refine the themes, drawing on the IFUM and PT theoretical frameworks for guidance. This approach ensured that our analysis was both theoretically grounded and open to emerging insights from the data. Phase 5 included reviewing and naming themes, with discrepancies resolved through consensus discussions among all the researchers. Results were validated through participant feedback in Phase 6, ensuring accuracy and resonance with their experiences, ultimately leading to the collaborative production of the final report through discussion and consensus among all authors.

Data analysis was assisted by MAXQDA Analytics Pro 2022 software.

### Rigor

The Criteria for Reporting Qualitative Research (COREQ) was used to report the study [30].

The trustworthiness [31, 32] of the study was established through transparent presentation of participant-generated results, while coherence in data analysis was achieved by detailing and verifying the reliability of intercoding analysis, ensuring that findings remained impartial and unbiased. Credibility [31] was upheld through content validation involving member checks with expert nurses and a nurse researcher, peer debriefing of results with participants, and sustained engagement. Data convergence was strengthened by triangulation among researchers [32]. Auditability [31] was maintained by thorough scrutiny of the audit trail report, ensuring comprehensive documentation of the research process and the decisions made throughout. To enhance transferability [31, 32], a clear and detailed description of the procedure and data analysis was provided, allowing readers to assess the study’s applicability in different settings.

### Ethical considerations

Ethical approval was obtained from the Ethics Committee of the Health Unit’s Innovation and Development of the hospital where the study was conducted. Nurses were provided with verbal and written information about the rationale of the study, data collection methods, and confidentiality in compliance with the Declaration of Helsinki. Written informed consent to participate was obtained from all participants before the interviews began. Code numbers were assigned to ensure participant anonymity (e.g. P1). After the completion of data collection, personal information was expunged from the transcripts to safeguard participant anonymity. The acquired data will be securely retained for a period of 10 years on a password-protected computer, adhering to the guidelines set forth by the Guide to the General Data Protection Regulation [33]. Subsequently, all data formats will be permanently deleted under the supervision of the research data support system.

## Results

Table 1 presents the participants’ characteristics, including their anonymized identifier, gender, professional role, and years of clinical experience in PACU.

The major theme, ‘uncertainty management strategies in clinical reasoning of nurses in PACU’, was drawn from the IFUM theoretical framework and further categorized into four themes based on the focus and descriptive characteristics of each strategy type: ignorance-focused strategies, uncertainty-focused strategies, response-focused strategies, and person-focused strategies. Subsequently, the PT framework was applied to identify the nursing realities that moderate the use of these strategies. The strategies were then categorized within the PT framework, which includes key nursing uncertainty-moderator realities, such as recipient, skills, setting, policies, goals, techniques, procedures, devices, agent, time sequence, happenings, and supportive relationships. Each of these nursing realities was integrated into the different strategies, meaning that not all realities are present in every strategy, but rather, they are tailored and aligned with specific strategies. Nonetheless, all these realities within each strategy ultimately converge toward the central purpose of nursing care.

**Table 1:** Characteristics of the participants

Participant ID	Gender	Professional Role	Clinical Experience (total, years)	Clinical Experience In PACU (years)
P1	Female	Nurse Practitioner	19	16
P2	Female	Registered Nurse	22	22
P3	Female	Nurse Practitioner	16	14
P4	Female	Nurse Practitioner	26	12
P5	Female	Registered Nurse	10	4
P6	Male	Nurse Practitioner	20	18
P7	Female	Registered Nurse	30	28
P8	Female	Registered Nurse	25	10
P9	Male	Registered Nurse	16	4
P10	Male	Registered Nurse	28	15
P11	Female	Registered Nurse	27	22
P12	Female	Registered Nurse	31	31
P13	Female	Registered Nurse	20	9
P14	Male	Nurse Pratictioner	23	19

**Table 2:** Ignorance-focused uncertainty management strategies

General Strategy	Specific Strategy		Illustrative Quotes
**Ignorance-focused**	**Recipient**	person centered care	‘You know that you must act in a certain way, there are protocols or guidelines, but they are not always suitable for the patients we have or the situation that lies ahead.’P13
		evidence based practice	‘Scientific preparation and theoretical-practical knowledge will have to be the basis for sustaining our actions. I run the risk of entering that line of reasoning, a little bit based only on experience, based on belief, which can give me some internal certainties and I can even believe that I am doing well but that is not in fact scientifically validated certainty.’P6
	**Skills**	postgraduate training	‘A very specific case and that stems from postgraduate training, has to do with respiratory assessment of patients, with auscultation, which was something that nurses used to resign because we think it is not our job. The analysis of a blood gas, for example. This is something that I think I am better at, and that I try to improve and understand what is going on with the patient.’ P10
		conflict management	‘Most of the problems that arise are exactly because of poor or no communication. It is an excellent resource. I put it at the bottom of the pyramid. Being worked on. If there is no communication, nothing can be done.’ P14
		technical-scientific communication	‘Learning how to structure your communication. To gather, structure and pass on that set of signs and symptoms in a technical and scientific way.’ P3
		critical thinking	‘Essentially, the strategy involves me having to ground myself more and have a critical vision of things. If I can understand, despite the strategy that I outlined was not the most correct one, I could not have the expected result, but I could identify what was at the origin of the poor planning of that strategy, I must work on these aspects.’ P14

**Table 3:** Uncertainty-focused management strategies

General Strategy	Specific Strategy		Illustrative Quotes
**Uncertainty-focused**	**Setting**	anticipatory preparation	‘When they are more critical situations like mechanical ventilated patients, more complicated surgeries, I try to prepare the unit to settling the patient. I prepare the invasive blood pressure and central venous pressure monitoring, and the endotracheal intubation material.’P1
		collaboration in managing patient handovers	‘That reasoning that ‘we need this one to leave because the patient coming in is at a higher risk level,’ can be very dangerous reasoning and can put patients at risk. It is not always easy to dismantle. Because a large part of the decisions does not pass through us or only through us. This generates some uneasiness precisely because at some moments we must allow events to unfold with some degree of uncertainty.’ P6
	**Policies**	creation of ‘led care’ protocols	‘Standardize protocols, review procedures, so that everyone can speak the same language and have the same line of thinking.’ P14
		discussion of standardized care practices	‘There should be opportunities to discuss occasional clinical cases to try to standardize care in those more occasional or unusual situations that may occur.’ P2
		continuous improvement projects	‘The primacy will be in terms of continuous improvement projects rather than exclusively in-service training. In-service training is important, but if it is not applied regularly and systematically, it is worthless. Continuous improvement projects, if they bring something new to our service, excellent!’ P9
		in-service team training	‘In-service training. Construction of working procedures. Regular training on the themes that make people more uncomfortable. Regular training is essential, even with themes suggested by the team.’ P3
		contingency planning	‘In case two or three major surgery patients arrive at the same time, if a patient becomes unstable or if there is a post-operative complication, there has to be a redistribution of staff to their units.’ P4
		workload adaptation	‘Sometimes you need to have availability. When you have these critical patients, you need to have time to be with them and be lucky enough to have certain details come to your attention. Because sometimes there is so much pressure, so many things to get everywhere, that we get a little lost. And there is no systematic evaluation, because there are always many requests, many interruptions, and this may go unnoticed.’ P2
		complying with schedules	‘To manage uncertainty, we should have fewer hours and fewer shifts in a row. I do not think anybody can think well or discern well, and the ones who get harmed are the patients. And the effort is greater.’ P12
	**Goals**	normalizing uncertainty	‘We are all in a constant learning process and therefore I think we should start looking at uncertainty differently.’ P9
		focusing on uncertainty	‘If you feel that you did not control the situation or that that situation is uncertain, you are going to create mechanisms, capacities, skills to control it, to make the uncertain less uncertain, to be able to better understand what happened or the phenomena. You are going to create conditions so that that situation would not be so uncertain anymore.’ P13
	**Techniques**	clinical case discussions	‘I find extremely relevant in uncertainty management to have regular team meetings where you can discuss and clarify doubts and uncertainties arising from clinical cases.’ P9
		simulated clinical practice exercises	‘Training, stimulating discussion of clinical cases, seeking simulation and doing clinical simulation on emergency events, anticipation of outbreaks of instability, prevention of post-anesthesia complications, pharmacology.’ P3
		systematizing patient assessments	‘I did a global assessment of the patient. We must see the patient as whole. You see the ABCDE assessment? The evaluation is by those parameters.’ P5
		outline the nursing process through record keeping	‘We often have a lot of turnovers and a lot of pressure, if we do not have things schematized, we end up making it easier. Nursing records help us refocusing. When I do an evaluation upon settling and discharge, I know that there are certain details that are not going to be forgotten, because I know that I want it to be recorded in the process that was evaluated. It forces schematization and evaluation and recording of that evaluation.’ P2
		debriefing	‘Most of the time, even working with other people around, our reasoning and our decision-making ends up being very solitary. That can indeed be disturbing, we may not be doing everything right. The point is that also because of what was debrief previously, I understand that we are all experiencing this difficulty.’ P6
		double-checking	‘You always end up discussing the various solutions and trying to find the right answer. It ends up being a shared decision, which will contribute to double checking in certain situations. It will reinforce the positive outcome.’ P2
		peer review	‘We are an open space unit, where the work is visible, where we are there in each other’s eyes and I easily anchor myself, exchange impressions and seek support with the colleagues who are there.’ P14
	**Procedures**	structured hand-offs	‘There is information that is given to us verbally when the operation room nurses are hand overing as we are setting up the patient in the unit. I think it is complicated. You absorb all the information they tell you, and then you lose information. It is ok that we can go online to check the information that is recorded but if there was a single process of handover, I think it would be easier.’ P5
		information systems	‘I think it is critical to have a greater connection to nursing information systems, like a system that would allow us to optimize the data that we could collect.’ P10
		collaborative multidisciplinary patient care plans	‘Small signs that we perceive deserve greater vigilance and attention. I try to validate with colleagues and trigger the anesthesiologist, so that we can jointly see of the sign that seems to me to highlight some risk for the patient. And try to somehow defend that that sign does not allow the patient to leave our surveillance.’ P6
	**Devices**	user-friendly clinical support software	‘In the other hospital where I worked, we already had the PICIS^®^, which was an extraordinary record system and allowed us to collect a lot of data. It makes our work much easier, and we have a greater focus on the patient.’ P10
		computing resources	‘And then there are the records and then you must take the information because the information is all in the computer and we only have two computers and then I do not know about therapeutic tables. Time is wasted.’ P7

### Ignorance-focused strategies

Several reported ignorance-focused strategies related to the recipient of nursing care and nurses’ skills. Table 2 shows the specific strategies, according to IFUM’s conceptual taxonomy, as well as the illustrative quotes.

### Uncertainty-focused strategies

Participants described different uncertainty-focused approaches, which aimed at logically structuring the uncertainty or disengaging from it, related to sub-themes of setting, policies, goals, techniques, procedures, and devices (cf., - Table 3).

### Response-focused strategies

Participants identified several response-focused strategies aimed at reducing or palliating their emotional distress from uncertainty, related to the sub-themes agent, time sequence and happenings (cf., - Table 4).

**Table 4:** Response-focused uncertainty management strategies

General Strategy	Specific Strategy		**Illustrative Quotes**
Response-focused	**Agent**	exercising self-reflectiveness	‘It is not about the patients; it is about me! These situations make me very anxious. It is also because of my personality, my way of being, my way of reacting to situations. Then at the end of the situations I try to stop, reflect, and realize if I did it right or not. Then I think: ‘I am glad nurse X was there to help me! It is always that uncertainty.’ P8
		exercising humility	‘I have to admit that the ideal in behavioral terms would be humility, the recognition of these limitations.’ P9
		emotion self-regulation	‘Even if you sit for 15 minutes, quietly reading a book, or doing nothing at all! May you concentrate, may you relax, may you strengthen yourself.’ P7
		humor	‘Then I have time to decompress, to use the humor that is always needed.’ P13
		acceptance of uncertainty: recognition of its fundamental nature	‘There must be some consultation out there that will help us to overcome these things, because sometimes uncertainty is not just a lack of knowledge. It has to do with our way of being, with our personality. Arrange some coping mechanisms to overcome this.’ P8
	**Time Sequence**	seeking personal space and distance	‘It is worrying and disappointing because we give our best every day here, and sometimes we just do not give more because the institution itself is so formatted that it conditions the person himself. From the physical structure, we still do not have an informal space for things of normal life.’P12
		optimizing working conditions (shifts < 12 h)	‘When we did a 12-hour shift, we needed some time for ourselves. When I do 12 hours, from then onwards at 6/7 my degree of concentration must be much more exhausting. I seem to get more tense because I am more afraid of failing. Because I am more tired, it seems that my brain takes longer to respond.’ P7
		timed work breaks	‘Work breaks should be a concern, not only for the unit but of the institution. In units like this it is surreal. It increases the clinical risk and the more we talk about this it seems that less and less is done.’ P12
	**Happenings**	positive cognitive reframing	‘I think it would be in everyone’s interest to also start to stop having that idea that meetings and talking about situations are always to harm those who were there, who intervened in the situation, and start to see this as an excellent source of information and even promoter of improvement in the quality and safety of care.’ P9

### Person-focused strategies

The strategies were mainly centered on developing supportive relationships (cf., Table 5).

**Table 5:** Person-focused uncertainty management strategies

General Strategy	Specific Strategy		Illustrative Quotes
**Person-focused**	**Supportive Relationships**	looking for help	‘I have already asked orthopedic nurses to help me with skin traction, which I did not know how to do or had no experience of doing. It is a risk to a patient to be doing something you do not know how to do. That is essential.’ P12
		cultivating reciprocity	‘We have a positive aspect in the team: we help each other, we work as a whole. That makes the uncertainties go smoothly. The team helps to mitigate the harm of uncertainty. In an emergency, the team comes together to tackle the situation that leads to such uncertainty.’ P1
		promoting collaborative communication	‘We can be comfortable to ask our questions, regardless of what it may bring to the other person. I believe that there must be a certain amount of comfort between us. It can be informal or formal, but with transparency. Of course, this is blurred by individual sensitivities.’ P6
		fostering stand up meetings	‘We should have small meeting moments to address certain situations that are linked to uncertainty in practice. To try to understand what failed you, what you did not do and what you should have done.’ P13
		team reflection exercises	‘In the face of uncertainty, you already have professional and personal experience. In similar situations, how did I act? I think that an important thing about working in intensive care units is to know and have the humility to ask for help. ‘Man, I am missing something here! Help me, please. What can I offer more?’ P4
		building open channels of communication	‘Provide moments of communication between the teams. Because we are on a small island, but we end up having some contact with the other and know what is going on in the other surgical services. Informally, we try to get some feedback on what happens, how people solve some situations. But this could be formalized at an institutional level. Of problem solving, of training plans, of projects. Of the error, for example.’ P13

**Fig. 1:**
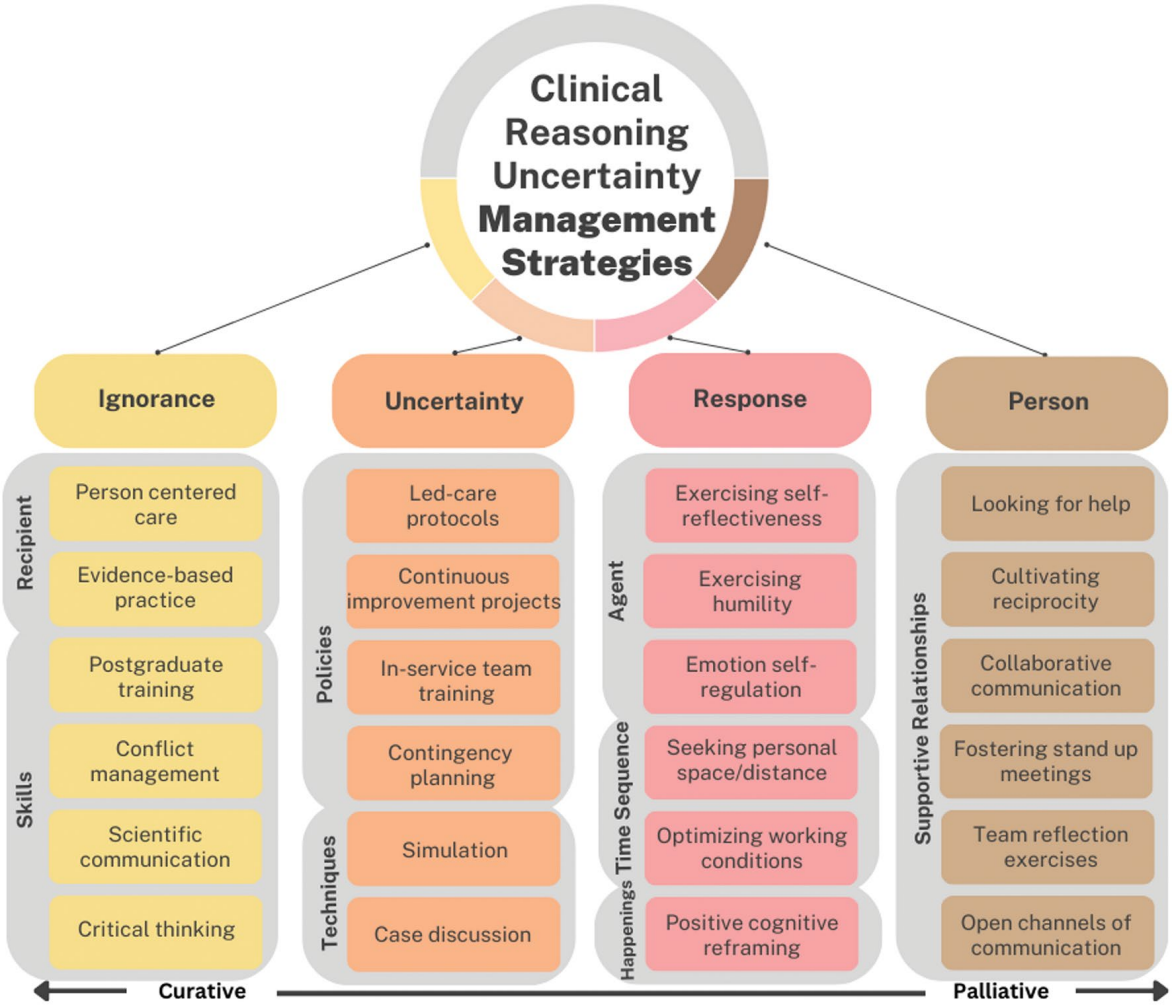
PACU nurses’ uncertainty management strategies

The main findings of the study are displayed in Fig. 1. As a result of the broad range of uncertainty management strategies used by nurses in their daily work at the PACU, the strategies widely described and their feasibility in the post-anesthesia context were highlighted. The grey areas in the picture are related with nursing realities.

## Discussion

The findings illustrate the diverse strategies nurses in the PACU setting use to manage uncertainty in clinical practice and shed further light on key needs and opportunities to improve the management of these uncertainties.

### Central purpose: curative versus palliative

Nurses employ probabilistic decision-making based on the differentiation of various cause-effect assumptions, while accounting to the realities that mediate situations and influence the central purpose of nurses’ clinical practice [13]. In this study, nurses demonstrated the prudence to orient themselves in ambiguous clinical situations and the flexibility to embrace multimodal uncertainty management strategies of curative and/or palliative intent. To address these issues, PACU nurses attempted to manage uncertainty using ignorance, uncertainty, response, and person-focused strategies. Strategies focused on ignorance and uncertainty were mainly centred on “curing” uncertainty by reducing ignorance or their consciousness of it. Response and person-focused strategies focus primarily on “palliating” responses to uncertainty, either by altering psychological responses to the triggering event or sharing the experience of uncertainty with others. Although the default strategy is to strive to cure the uncertainty, limited scientific knowledge and the psychological burdens of uncertainty require healthcare providers to also pursue its palliation [17]. However, uncertainty management strategies often intersect and overlap in their goals, which highlights the complexity of the phenomenon and the importance of their personal significance.

### Ignorance-focused uncertainty management strategies

In complex clinical systems, with nonlinear dynamics characterized by a spectrum of (ir)reducible outcomes and (in)determinations, uncertainty can be a positive force. Namely, it might prompt clinical imagination through reflective questioning and use of active awareness of multiple possibilities to support lateral thinking in the clinical reasoning process [34]. Ignorance-focused strategies aim to overcome nescience. The growing complexity of patient care requires proficiency in information seeking, and the identification of highly reliable evidence [35]. In this study, PACU nurses emphasized uncertainty management strategies focused on the recipient and their skills, that helped them reduce ignorance and provide comprehensive evidence-based care.

### Uncertainty-focused management strategies

Uncertainty is a universal attribute of science, which expands in proportion to the extension of knowledge [36]. Epistemic uncertainty concerns phenomena that we do not currently know but that we would be able to know, at least in theory, i.e., the knowledge on which decision-making is grounded stems from epistemic uncertainty [34]. In this study, participants utilized various uncertainty-focused strategies that aim to provide clarity and structure to their uncertainty, thereby enabling them to approach it in a more intentional and organized manner. ‘In-service team training’ and ‘simulated clinical practice exercises’ intersected with ignorance-focused strategies. Participants described these approaches in terms of knowledge acquisition as well as disengagement from their ignorance. Participants identified that protocol-led care, discussion of standardized care practices, continuous improvement projects, in-service team training, simulated clinical practice exercises, and debriefing, are common policy-oriented strategies for ordering uncertainty. Other medical uncertainty-focused strategies like maximizing attention (e.g., double checking, peer review) and minimizing attention (e.g., systematizing patient assessments) are also meaningful. Importantly, all these strategies may reduce uncertainty in clinical reasoning, but they may also encourage cognitive passivity among nurses. This may especially be the case when there is a lack of time and resources to critically appraise and synthesize the best available evidence, or a lack of mentors to assist in the knowledge search strategy [12, 35].

### Response-focused uncertainty management strategies

Uncertain contexts can cause individuals to feel threatened by a lack of sense of belonging or lack of control [37], which can trigger compensatory strategies to adapt themselves [38]. In this sense, nurses’ personality characteristics prompt different response-focused strategies to respond to uncertainty due to distinct needs [39]. Because feeling uncertain about oneself is generally aversive and maladaptive, our participants were motivated to reduce self-uncertainty through self-reflectiveness, exercising humility, emotion self-regulation, and humor. Nurses’ self-evaluation appears to relate positively to their clinical decision-making: nurses with high self-esteem, self-efficacy, and internal locus of control make autonomous intervention decisions, seek alternative options, analyze patient data in detail, evaluate the consequences of these interventions, and partially seek new and additional information [40]. Also, active self-awareness allows nurses to attend to their cognitive, affective, and behavioral selves, which can improve their caring behaviors and sense of well-being [41].

In addition, adopting a new response-focused mindset and positive cognitive reframing reduces occupational stress and restores physical, mental, and spiritual nurses’ well-being, enabling the provision of high quality [42]. Conjointly, research [43] confirms the effectiveness of humor interventions in promoting the meaningfulness and work enjoyment. Moreover, the response-focused strategies reported by study participants integrate moments of conscious engagement with uncertainty through moments of passive breaks from work that allows for self-regulation of emotions and reduction of work fatigue, which ultimately help to reduce anxiety and mitigate negative responses to uncertainty [44, 45]. These primary palliative strategies for handling irreducible uncertainty illustrate the mindset shift that allows nurses to accept the reality and irreducibility of uncertainty, and to orient themselves towards it in a more flexible and self-forgiving way.

### Person-focused uncertainty management strategies

This study also showed how interpersonal and interprofessional relationships can be a resource to cope with uncertainty. Participants reported that person-focused uncertainty management strategies, such as building supportive and collaborative relationships with colleagues and sharing experiences of uncertainty with peers, promoted mutual trust and support among nurses, reduced feelings of isolation in decision-making, and lightened the emotional burden of uncertainty. Many participants stated that they anchored themselves to team members in situations of uncertainty where immediate decision-making was demanded. Yet nurses may also incorporate misinformation into the clinical reasoning process through passive evaluation of the accuracy of evidence and exposure to knowledge negligence [46]. While reliance on colleagues may be beneficial, it is thus not necessarily the primary goal of collaborative work, because there is a difference between sharing uncertainty and sharing professional responsibility either to know key information, or to care for the patient. The growing trend of interprofessional practices has led to an increasing focus on interprofessional collaboration [47]. Participants’ reported uncertainty management strategies, such as seeking help, cultivating reciprocity, promoting collaborative communication, fostering stand up meetings, team reflection exercises and building open communication channels, imply a more fundamental goal of interprofessional collaboration. Working collaboratively is a way to better manage uncertainty: it fosters an ownership mindset, negotiation of overlapping roles and tasks, and creation of spaces to build caring relationships. The collaborative management of uncertainty can ultimately promote more synergistic, efficient, safe and high-quality healthcare delivery [47, 48].

### Limitations

While data collection limited to one PACU might be viewed as a study limitation, detailed descriptions and contextual exploration ensured transferability, enabling readers to assess applicability in diverse contexts. Furthermore, contextual exploration incorporating the institutional mandate, and nursing conceptual framework facilitated broader interpretations.

Conducting interviews within the hospital may have influenced participants’ responses due to its workplace association. To minimize this, interviews were held in a private room outside the clinical setting, ensuring a neutral, distraction-free, and convenient environment for open discussions. Having an insider researcher familiar with the local reality enhances problem-solving by leveraging participants’ insights. Also, the dual role presented opportunities to address ethical and practical concerns, including the longitudinal approach fostering rapport, trust, and familiarity with participants, while the hazarding process minimized bias and clarified inconsistencies between narrative and behavior.

### Conclusions and recommendations

The study aimed to describe and map the uncertainty management strategies employed by nurses in PACU within two theoretical frameworks, aligning them with the core goals of nursing care. These strategies serve as practical tools, not merely theoretical concepts, helping nurses navigate situations with high uncertainty. More work is needed to further explore how to improve these strategies and the ability of nurses and other healthcare providers to manage clinical uncertainty more effectively. These are necessary skills to achieve the ultimate outcomes of improving patient safety and achieving high-quality, cost-effective care.

Uncertainty in clinical reasoning should be understood as a complex, multi-dimensional phenomenon, with different types that have overlapping and intersecting features and manifestations. A crucial dimension of knowledge and action in healthcare settings is to accept a certain degree of uncertainty as inherent to practice. It also is important to admit the influence of cognitive biases in clinical reasoning that do not necessarily undermine safety of care but may be a crucial nudge toward a more systematic approach to managing uncertainty.

The IFUM conceptual taxonomy allowed us to systematically approach uncertainty management strategies used by PACU nurses. The range of strategies promotes an understanding of uncertainty management as a dynamic metacognitive process of self-regulation and adaptation. However, the strategies listed are purely descriptive as they do not provide definitive answers to the normative question of uncertainty management. An unmet future research need is for a Prescriptive Theory frame that can provide guidance on what uncertainty management strategies should be used to achieve defined goals for planned activities, and to help nurses achieve an adaptive balance between cure and palliation of uncertainty. Conceptual frameworks such as the IFUM can facilitate this work by enabling the uncertainty management strategies used by nurses to be logically organized and approached.

## Electronic supplementary material

Below is the link to the electronic supplementary material.

Supplementary Material 1

## Data Availability

Data that support the findings of this study are available from the corresponding author upon reasonable request.
