# Supplementary material for: Uncertainty management strategies in clinical reasoning: perceptions of nurses in post-anesthesia care units
Source: BMC Nurs. 2025 May 28;24:605. doi: 10.1186/s12912-025-03193-8 (PMC12117670; doi:10.1186/s12912-025-03193-8)
Supplement: Supplementary file 1 — Supplementary Material 1 [file 12912_2025_3193_MOESM1_ESM.docx]

**Supplementary File 1.** Interview moderator guide

| **Questions** |
| --- |
| 1. Describe a clinical experience from a scenario/situation of uncertainty where you felt you made a difference. a) What information/data did you use to make decisions? b) How did you guide nursing clinical reasoning? |
| 2.When you experienced situations of uncertainty, how did you try to solve the problem? A) How did you identify possible solutions? b)What was the result of this decision? c) Reflecting on the situation, do you think there would have been alternative strategies? d) Do you think that these alternative strategies could have changed the outcome of the situation? In what way? |
| 3. What resources do you know and use to manage uncertainty in nursing clinical reasoning? Describe how you use these resources. |
